# A case–case study on the effect of primary and booster immunization with China-produced COVID-19 vaccines on prevention of pneumonia and viral load among vaccinated persons infected by Delta and Omicron variants

**DOI:** 10.1080/22221751.2022.2103455

**Published:** 2022-08-05

**Authors:** Dan Wu, Ying Ye, Lin Tang, Ai-Bin Wang, Rui Zhang, Zhao-Hui Qian, Fu-Zhen Wang, Hui Zheng, Chang Huang, Xiao-Ya Lv, Hai-Feng Wang, Yan-Yang Zhang, Jing-Jing Pan, Ya-Fei Li, Ming-Xia Lu, Chang-Shuang Wang, Ya-Ting Ma, Zhi-Jie An, Lance Everett Rodewald, Zun-Dong Yin, Xuan-Yi Wang, Zhi-Yin Wu, Yi-Ming Shao

**Affiliations:** a National Immunization Program, Chinese Center for Disease Control and Prevention, Beijing, People’s Republic of China; b Henan Provincial Disease Control and Prevention, People’s Republic of China, Henan; c Beijing Ditan Hospital, Capital Medical University, Beijing, People's Republic of China; d National Center for Clinical Laboratories, Beijing Hospital, National Center of Gerontology, Institute of Geriatric Medicine, Chinese Academy of Medical Sciences, Beijing, People’s Republic of China; e NHC Key laboratory of Systems Biology of Pathogens, Institute of Pathogen Biology, Chinese Academy of Medical Sciences and Peking Union Medical College, Beijing, People’s Republic of China; f Chinese Field Epidemiology Training Program, Chinese Center for Disease Control and Prevention, Beijing, People's Republic of China; g Nanning Center for Disease Control and Prevention, Guangxi, People's Republic of China; h Development Center for Medicine and Science &Technology, National Health Commission, People’s Republic of China, Beijing; i Key Laboratory of Medical Molecular Virology of MoE & MoH, Shanghai Institute of Infectious Disease and Biosecurity, Shanghai, People’s Republic of China; j Institutes of Biomedical Sciences, Shanghai Medical College, Fudan University, Shanghai, People’s Republic of China; k State Key Laboratory of Infectious Disease Prevention and Control, National Center for AIDS/STD Control and Prevention, Chinese Center for Disease Control and Prevention, Beijing, People’s Republic of China

**Keywords:** COVID-19 vaccine, Ct value, case-case study, real-world study, booster immunization

## Abstract

Using a three-prefecture, two-variant COVID-19 outbreak in Henan province in January 2022, we evaluated the associations of primary and booster immunization with China-produced COVID-19 vaccines and COVID-19 pneumonia and SARS-CoV-2 viral load among persons infected by Delta or Omicron variant. We obtained demographic, clinical, vaccination, and multiple Ct values of infections ≥3 years of age. Vaccination status was either primary series ≥180 days prior to infection; primary series <180 days prior to infection, or booster dose recipient. We used logistic regression to determine odds ratios (OR) of Delta and Omicron COVID-19 pneumonia by vaccination status. We analysed minimum Ct values by vaccination status, age, and variant. Of 826 eligible cases, 405 were Delta and 421 were Omicron cases; 48.9% of Delta and 19.0% of Omicron cases had COVID-19 pneumonia. Compared with full primary vaccination ≥180 days before infection, the aOR of pneumonia was 0.48 among those completing primary vaccination <180 days and 0.18 among booster recipients among these Delta infections. Among Omicron infections, the corresponding aOR was 0.34 among those completing primary vaccination <180 days. There were too few (ten) Omicron cases among booster dose recipients to calculate a reliable OR. There were no differences in minimum Ct values by vaccination status among the 356 Delta cases or 70 Omicron cases. COVID-19 pneumonia was less common among Omicron cases than Delta cases. Full primary vaccination reduced pneumonia effectively for 6 months; boosting six months after primary vaccination resulted in further reduction. We recommend accelerating the pace of booster dose administration.

## Introduction

As of 21 February 2022, World Health Organization (WHO) data showed that there were nearly 500 million COVID-19 cases and over 6 million deaths reported globally [1]. COVID-19 vaccines are an important means for preventing COVID-19 and its related morbidity and mortality. Real-world study of COVID-19 vaccines, at home and abroad, has been showing that while none of the available COVID-19 vaccines completely prevent COVID-19 infection, all are significantly effective for preventing hospitalization, severe illness, and death caused by severe acute respiratory syndrome coronavirus 2 (SARS-CoV-2) infection [2–5]. Emergence of new variants of concern demands continuous evaluation of vaccines and their impact. WHO listed the B.1.1.529 (Omicron) variant as a Variant of Concern (VOC) on 26 November 2021, and since then, Omicron rapidly became the globally dominant variant due to its higher transmissibility and partial immune escape, although with lower virulence compared with the ancestral strain and other VOCs [6–8]. China has adopted the strict epidemic prevention and control measures of “external prevention of importation, internal prevention of rebound,” leading to low levels of local transmission, with all local transmission being associated with importation outbreaks. In 2021, most COVID-19 outbreaks were caused by the Delta variant, and in 2022, most have been Omicron outbreaks.

Important knowledge gaps are whether primary and booster immunization with China-produced COVID-19 inactivated vaccines can reduce disease severity caused by the new variants and whether they can reduce the viral load during infection. Population immunity thus far in China is only vaccine-induced due to the paucity of local transmission that would lead to hybrid immunity. One study in Jiangsu showed that primary vaccination and booster doses using China-produced inactivated COVID-19 vaccines reduced risk of Delta infection progressing from mild to severe and critical severe COVID-19 [9].

In January 2022, a local Delta-variant outbreak occurred in Zhengzhou and Xuchang Yuzhou (hereinafter referred to as “Yuzhou”) in Henan province. Coincidentally, a local Omicron outbreak occurred in Anyang city, which is also in Henan Province. The simultaneous outbreaks provided an opportunity to evaluate associations between primary and booster immunization and pneumonia among persons infected with Delta or Omicron variants, and to assess association of vaccination and viral load during infection. We reported our evaluations.

## Methods

### Setting and outbreak

The setting was three cities in Henan province – Zhengzhou, Yuzhou, and Anyang. As of 1 January 2022, prior to the outbreak, full-series primary vaccination coverage levels were 87.22%, 94.67%, and 80.28% of whole populations in Zhengzhou, Yuzhou, and Anyang, respectively. The vaccines used in the outbreak setting included two inactivated vaccines (91.80% of doses), BBIBP-CorV, produced by Sinopharm and CoronaVac, produced by Sinovac; one recombinant protein vaccine (7.53% of doses), ZF2001, produced by Anhui Zhifei Longcom; and one adenovirus type-5 (Ad5)-vectored vaccine (0.67% of doses), injectable Convidecia, produced by CanSinoBio. During the time of the Henan outbreak, COVID-19 antivirals were not licensed or in use.

On 2 January 2022, a ceramics factory worker in Yuzhou city was diagnosed with SARS-CoV-2 infection after a routine PCR test prior to hospital admission for surgery came back positive. As an investigation progressed, additional infections were detected in Yuzhou and Zhengzhou city (the Zheng-Yu transmission chain). On 4 January, lock down was implemented hierarchically to block transmission in the two cities. The Zheng-Yu transmission chain lasted until January 19 and ultimately consisted of 500 Delta (B.1.617.2) strain infections.

On 8 January 2022, an employee of a medical device company and a middle school student were confirmed PCR positive for SARS-CoV-2 infection when seeking health care in Anyang city hospitals (the Anyang transmission chain). Two days later, lock-down was implemented hierarchically to block transmission in the communities and the middle school. Eventually, 468 Omicron (B.1.1.529.1) infections were identified within the Anyang transmission chain, which lasted until January 29.

### Study design and data sources

We used a case-case study [10] design to evaluate the association between vaccination and pneumonia among persons infected with Delta or Omicron variants. We used descriptive statistics to analyse the minimum Ct value obtained during the time between the first RT–PCR positive test and the end of hospitalization (a proxy for maximum viral load) among infected individuals by age, variant, and vaccination status, explore the association of vaccination and PCR (RT–PCR) cycle threshold (Ct) values in Delta and Omicron infections.

#### SARS-CoV-2 infections

We obtained data from the National Notifiable Disease Reporting System (NNDRS) on all individuals ≥3 years old diagnosed with SARS-CoV-2 infection. Data included age, gender, identity card (ID) number (for linking across data bases), and the most severe clinical classification between 2 and 23 January. The subjects in our study were close contacts of laboratory-proved infections and therefore all had known exposure to SARS-CoV-2.

#### Immunization histories

We obtained vaccination records from the National Immunization Information System, linked through infection ID to national ID, which indexes the immunization information system.

#### PCR tests

At least two different PCR test kits, approved by China’s National Medical Products Administration (i.e. BioGerm test kit, bioPerfectus Technologies test kit, Ediagnosis test kit, DAAN GENE test kit, XABT test kit, or Sansure Biotech test kit), were used to confirm RT–PCR positivity of each person infected. We obtained all PCR test results from local CDCs and hospitals including infection ID, sample source, test kit manufacturer, Ct value of ORF1ab and N targets, and sample collection date. To ensure that the Ct values were evaluable in our subject-level CT-value analyses, we excluded subjects for which Ct N-target values exist but Ct ORF1ab-target values were 0 or null, <10, or ≥40; subjects for which Ct ORF1ab values existed but Ct N values were 0 or null, <10, or ≥40; and subjects for which the absolute value of the difference between Ct (ORF1ab) and Ct (N) was ≥5.

#### Variant classification

Based on gene sequencing reports from the provincial and national laboratories, the Zheng-Yu transmission chain was caused by Delta (B.1.617.2) and the Anyang transmission chain was caused by Omicron (B.1.1.529.1).

### Definitions

#### Vaccination status

Based on vaccination history, individuals were classified into one of three mutually-exclusive categories: those who received full primary vaccination <180 days before diagnosis, those who received full primary vaccination ≥180 days before diagnosis, and those who had a booster dose. Full primary vaccination consisted of completion of two doses of an inactivated vaccine or one dose of the Ad5-vectored vaccine or three doses of the CHO protein recombinant vaccine 14 days or more before exposure to SARS-CoV-2, or receipt of one booster dose but within seven days of exposure. Booster dose receipt consisted of those who completed 3 doses of inactivated vaccine or 2 doses of Ad5 vaccine at least seven days prior to exposure. There was a small number (69) of individuals with the unusual vaccination status of unvaccinated or partially vaccinated; these individuals were not included in the analyses.

#### Severity and outcomes

Asymptomatic cases were individuals who never developed any symptoms of any type during quarantine. Mild cases had one or more subjectively mild symptoms of infection, but did not have any signs of pneumonia on imaging. Moderate cases had manifestations of pneumonia along with any symptoms of COVID-19 such as fever, cough, or other respiratory symptoms. Diagnosis of pneumonia was based on clinical symptoms and CT imaging. Chest CT imaging findings suggestive of COVID-19 pneumonia were multiple bilateral ground glass opacities, often rounded in morphology, with peripheral and lower lung distribution [11].

Severe cases met any of the following criteria: (a) respiratory distress or respiratory rate 30 breaths/minute or more, (b) oxygen saturation less than 93% at rest, (c) a ratio of arterial partial pressure of oxygen (PaO2) to fractional inspired oxygen concentration (FiO2) of <300 mmHg (with adjustment for altitudes above 1000 metres), or (d) patients with pneumonia having >50% lesion progression within 24–48 h seen in pulmonary imaging. Critical Cases met any of the following criteria: (a) respiratory failure or need for mechanical ventilation, (b) shock, or (c) other organ failure that required monitoring and treatment in the ICU.

### Statistical analysis

For multivariate analysis of the associations between the three vaccination statuses (full primary vaccination ≥180 days and no booster dose, full primary vaccination <180 days, booster dose) and the three main outcomes (symptomatic COVID-19, pneumonia, and severe/critical COVID-19), outcomes were dependent variables, vaccination statuses were independent variables, and gender, age group (3–17 years old, 18–49 years old, and ≥50 years old), and presence of underlying disease(s) were considered potentially confounding variables. The age groupings were selected to reflect differences in vaccine recommendations and distribution of comorbidities: booster doses were not recommended for the youngest group; 95% of comorbidities were concentrated in the oldest group. To ensure common-direction assessments of vaccine effectiveness, we used the theoretically least protective vaccination status – full primary vaccination ≥180 days and no booster dose – as the reference group. We used conditional logistic regression-determined odds ratios (ORs) and adjusted ORs (aORs) of pneumonia to compare the reference group to the full primary vaccination <180 days group and the booster group, stratified by gender, age group, and presence of underlying conditions. Reductions in outcome risk were calculated by 1-aOR. For the minimum Ct value analysis, we obtained all Ct values for infected individuals and determined the minimum Ct value for each person of their multiple consecutive nucleic acid tests. We compared minimum Ct values in the vaccination groups stratified by age group after matching individuals by PCR reagent manufacturer. We used the Kruskal Wallis rank sum test to assess statistical significance of differences in minimum Ct value by vaccination group. SAS (version 9.4; SAS Institute) was used for matching and statistical analyses.

### Ethical review

COVID-19 is a Level-two infectious disease being managed as a Level-one (highest) infectious disease. Investigation of outbreaks is a responsibility of the institutions with which the authors are affiliated. China CDC and the CDC system have access to individual-level disease and vaccination data in their routine jobs. Analytic data sets were de-identified. Ethical Review Committee approval for this routine public health work is not required.

### Role of the funding source

Investigators and authors were employed by the funder, and therefore, the funder participated in all aspects of the study, including conceptualization, design, data collection, analysis, interpretation, drafting the manuscript, and the decision to submit the manuscript for publication.

## Results

**Figure 1.**
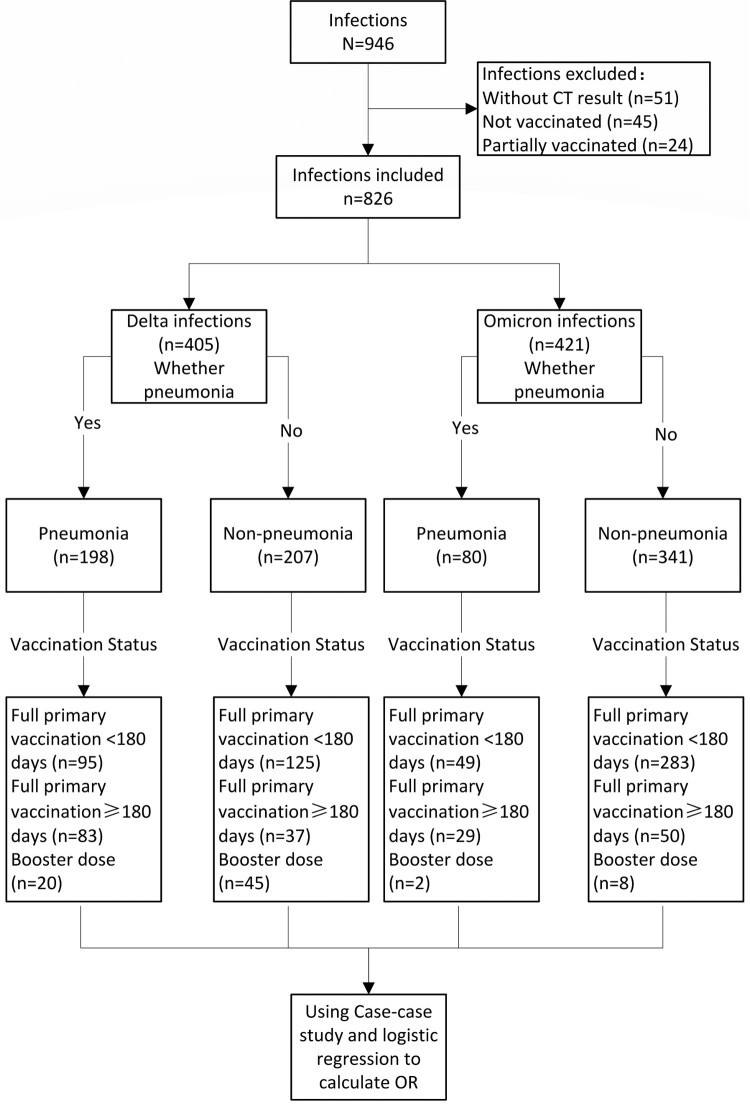
Case-case study flow chart and study subjects.

During the study period in the study setting, there were 946 individuals aged 3 years or older who were diagnosed by PCR as having SARS-CoV-2 infection. There were 69 infected individuals who were unvaccinated or partially vaccinated, and among them, 21 (30.43%) had underlying conditions. There were 826 infected individuals with CT imaging results who had completed full primary vaccination or received a booster dose and who were, therefore, eligible for the case-case study: 405 were infected by Delta and 421 were infected by Omicron (Figure 1). The male: female ratios for Delta and Omicron infections were 1:1.37 and 1:1.31, respectively, and 48.89% and 19.00% of Delta and Omicron infections, respectively, met COVID-19 pneumonia criteria (Table 1). All individuals under 18 years old had received full primary vaccination <180 days prior to infection. Table 2 shows the distribution of pneumonia by age group and vaccination status.

**Table 1.** Demographic characteristics, vaccination status, and clinical classification of the case-case study subjects, Henan, 2 January 2022–23 January 2022.

Variable	*n* (%)		
	Delta Infection *N* = 405	Omicron Infection *N* = 421	Total *N* = 826
Gender			
Female	234 (57.8)	239 (56.8)	473 (57.3)
**Male**	171 (42.2)	182 (43.2)	353 (42.7)
**Age Group (years)**			
**<18**	60 (14.8)	194 (46.1)	254 (30.8)
**18–49**	175 (43.2)	172 (40.9)	347 (42.0)
≥50	170 (42.0)	55 (13.0)	225 (27.2)
Vaccination Status			
Full primary vaccination <180 days	220 (54.3)	332 (78.9)	552 (66.8)
Full primary vaccination ≥180 days	120 (29.6)	79 (18.7)	199 (24.1)
Booster vaccination	65 (16.1)	10 (2.4)	75 (9.1)
Vaccine Type			
Inactivated vaccine	374 (92.4)	403 (95.7)	777 (94.1)
CHO vaccine	29 (7.1)	16 (3.8)	45 (5.4)
Ad5 vaccine	2 (0.5)	2 (0.5)	4 (0.5)
Comorbidities			
No	352 (86.9)	389 (92.4)	741 (89.7)
Yes	53 (13.1)	32 (7.6)	85 (10.3)
Pneumonia			
No	207 (51.1)	341 (81.0)	548 (66.3)
Yes	198 (48.9)	80 (19.0)	278 (33.7)
Clinical Severity			
Asymptomatic	19 (4.7)	37 (8.8)	56 (6.8)
Mild	186 (45.9)	304 (72.2)	490 (59.3)
Moderate	195 (48.2)	80 (19.0)	275 (33.3)
Severe	3 (0.7)	0	3 (0.4)
Critical Severe	2 (0.5)	0	2 (0.2)

**Table 2.** COVID-19 pneumonia, stratified by age group, vaccination status and VOC, Henan, 2 January 2022–23 January 2022.

Age Group (years)	Pneu-monia	Full primary vaccination ≥180 days *n* (%)		Full primary vaccination <180 days *n* (%)		Booster dose *n* (%)	
		Delta	Omicron	Delta	Omicron	Delta	Omicron
3–17	No	-	-	56 (93.3)	168 (86.6)	-	-
	Yes	-	-	4 (6.7)	26 (13.4)	-	-
18–49	No	27 (39.7)	35 (66.0)	36 (53.7)	98 (88.3)	33 (82.5)	6 (75.0)
	Yes	41 (60.3)	18 (34.0)	31 (46.3)	13 (11.7)	7 (17.5)	2 (25.0)
≥50	No	10 (19.2)	15 (57.7)	33 (35.5)	17 (63.0)	12 (48.0)	2 (100.0)
	Yes	42 (80.8)	11 (42.3)	60 (64.5)	10 (37.0)	13 (52.0)	0
Total	No	37 (30.8)	50 (63.3)	125 (56.8)	283 (85.2)	45 (69.2)	8 (80.0)
	Yes	83 (69.2)	29 (36.7)	95 (43.2)	49 (14.8)	20 (30.8)	2 (20.0)

### Pneumonia by variant and vaccination status

The percent of Delta variant infections that were pneumonia ranged by vaccination group from 30.77% to 69.17%, and the percent Omicron infections that were pneumonia ranged from 14.76% to 36.71%. Among people ≥50 years old who had full primary vaccination ≥180 days before Delta infection, 80.77% had pneumonia. Among people ≥50 years old who had full primary vaccination ≥180 days before Omicron infection, 42.31% had pneumonia (Table 2).

### Factors associated with pneumonia

Table 3 shows associations between gender, age group, comorbidities, and vaccination status and pneumonia for Delta and Omicron cases. Univariate and multivariate analyses showed that variant, age group, and vaccination status were statistically significantly associated with pneumonia. Multivariate analysis showed that compared with Delta, the odds ratio (OR) of association with pneumonia in the Omicron group was 0.34 (95%CI: 0.24–0.48).

**Table 3.** Factors associated with Delta and Omicron variant pneumonia.

Variable		Delta variant				Omicron variant				
	Pneumonia	OR	*p* value	Adjusted OR	*p* value	Pneumonia	OR	*p* value	Adjusted OR	*p* value
	N (%)	(95% CI)		(95% CI)		N (%)	(95% CI)		(95% CI)	
Gender										
Female	122(52.1)	Ref		Ref		44(18.4)	Ref		Ref	
Male	76(44.4)	0.73 (0.49–1.09)	0.127	0.72 (0.46–1.13)	0.153	36(19.8)	1.09 (0.67–1.78)	0.723	1.27 (0.76–2.14)	0.359
Age Group (years)										
<18	4(6.7)	Ref		Ref		26(13.4)	Ref		Ref	
18–49	79(45.1)	11.52 (4.00–33.15)	<0.0001	10.16(3.39–30.43)	<0.0001	33(19.2)	1.53 (0.88–2.69)	0.135	0.99 (0.5–1.89)	0.963
≥50	115(67.7)	29.27 (10.10–84.80)	<0.0001	25.70(8.56–77.10)	<0.0001	21(38.2)	3.99 (2.02–7.90)	<0.0001	1.45 (0.56–3.63)	0.433
Comorbidities										
No	163(46.3)	Ref		Ref		65(16.7)	Ref		Ref	
Yes	35(66.0)	2.25 (1.23–4.13)	0.001	1.33 (0.67–2.65)	0.423	15(46.9)	4.40 (2.09–9.25)	<0.0001	2.95 (1.16–7.63)	0.024
Vaccination Status										
Full primary ≥180 days	83(69.2)	Ref		Ref		29(36.7)	Ref		Ref	
Full primary <180 days	95(43.2)	0.34 (0.21–0.54)	<0.0001	0.48 (0.28–0.81)	0.006	49(14.8)	0.30 (0.17–0.52)	<0.0001	0.34 (0.17–0.67)	0.002
Booster dose	20(30.8)	0.20 (0.10–0.38)	<0.0001	0.18 (0.09–0.35)	<0.0001	2(20.0)	–	–	–	

Note: *Indicates that the number of cases is too small to make a valid estimate.

With analyses restricted to Delta infections, univariate analysis showed that age group, presence of one or more comorbidities, and vaccination status were statistically significantly associated with pneumonia. For Delta infections by age group, compared with <18 years old, the ORs of having COVID-19 pneumonia among 18–49 years and ≥50 years were 11.52 (95%CI: 4.00–33.15) and 29.27 (95%CI: 10.10–84.80), respectively, and compared with full primary vaccination ≥180 days, the ORs of having pneumonia among full primary vaccination <180 days and booster dose groups were 0.34 (95%CI: 0.21–0.54) and 0.20 (95%CI: 0.10–0.38), respectively (Table 3).

Multivariate analysis among Delta variant infections showed that age group and vaccination status were statistically significantly associated with pneumonia. Compared with <18-year-olds, the OR of having pneumonia among the ≥50 years age group was 25.70 (95%CI: 8.56–77.10). Compared with full primary vaccination ≥180 days, the ORs of having pneumonia among primary vaccination ≤180 days and booster dose groups were 0.48 (95%CI: 0.21–0.64) and 0.18 (95%CI: 0.09–0.35) (Table 3).

With analyses restricted to Omicron infections, univariate analysis showed that age group, presence of comorbidities, and vaccination status were statistically significantly associated with pneumonia. Compared with <18-year-olds, the OR of having pneumonia among ≥50-year-olds was 3.99 (95%CI: 2.02–7.90). Compared with no comorbidities, the OR of having pneumonia among those with one or more comorbidities was 4.40 (95%CI: 2.09–9.25). Compared with full primary vaccination ≥180 days, the OR of having pneumonia among those with primary vaccination ≤ 180 days was 0.30 (95%CI: 0.17–0.52) (Table 3).

Multivariate analysis among Omicron infections showed that comorbidities and vaccination status were statistically significantly associated with pneumonia. Compared with no comorbidities, the OR of having pneumonia among those with one or more comorbidities was 2.95 (95%CI: 1.16–7.63). Compared with full primary vaccination ≥180 days, the OR of having pneumonia among those with primary vaccination ≤ 180 days was 0.34 (95%CI: 0.17–0.67) (Table 3).

### Viral load (Ct value analysis)

**Figure 2.**
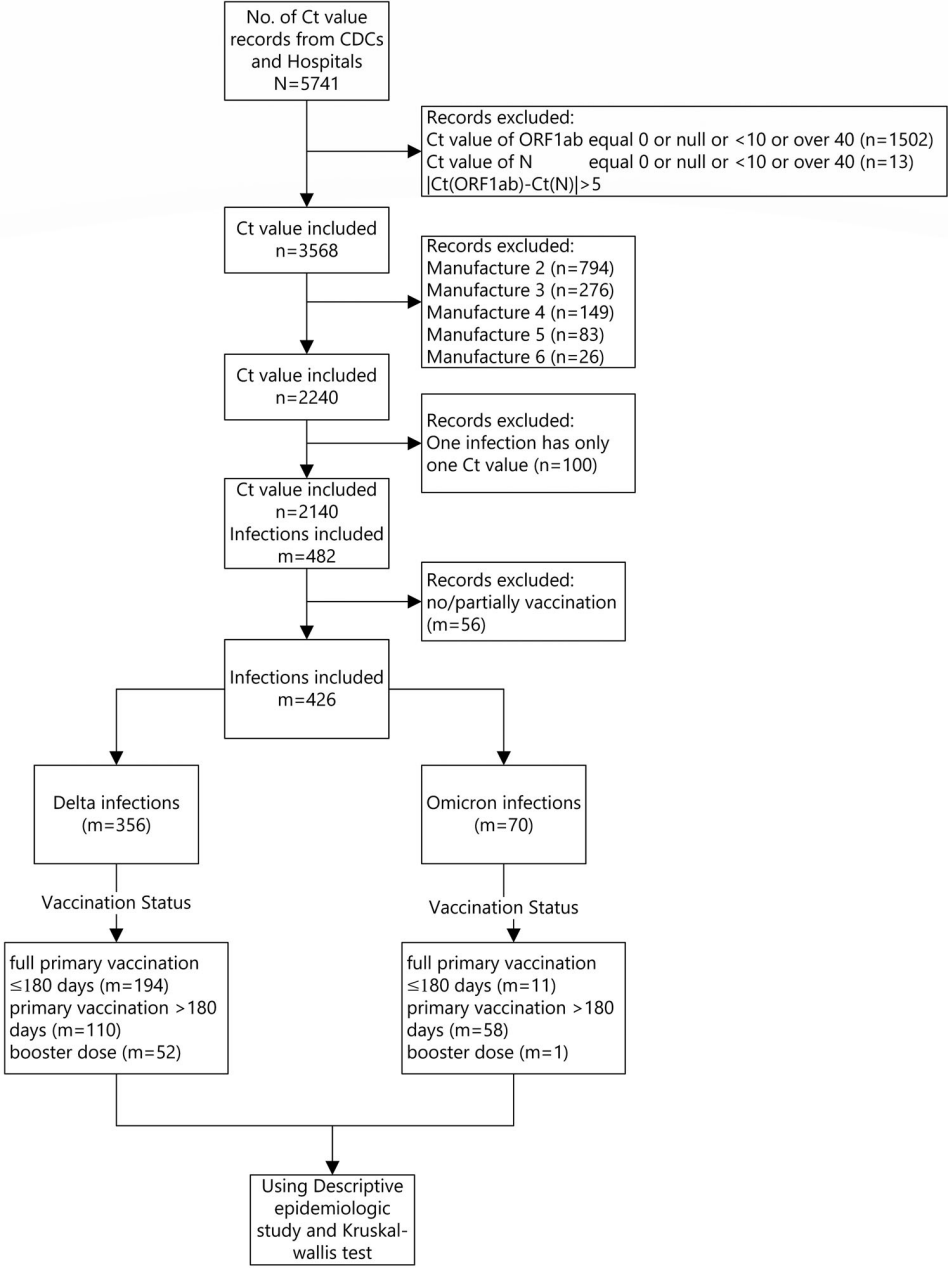
Minimum Ct value study subjects.

There were 2140 samples that met all inclusion and no exclusion criteria and were included in the Ct value analysis. Among the 946 infected individuals, 426 used the same PCR manufacturer reagent, 356 were Delta variant infections and 70 were Omicron infections (Figure 2).

Among those infected with the Delta variant, the medians of the minimum Ct values (ORF1ab target) among the primary vaccination ≥180 days group, the primary vaccination <180 days, and the booster dose group were 21.7, 22.0, and 23.7, respectively. The medians of the minimum Ct values (N target) were 20.9, 21.0, and 22.0, respectively. There were no statistically significant differences.

Among Omicron variant infections, the medians of the minimum Ct value (ORF1ab target) in the primary vaccination ≥ 180 days group and the primary vaccination <180 days group were both 23.0; the minimum Ct values (N target) were consistent with Ct values (ORF1ab target); there was no significant difference between groups. Since there was only one data-eligible infection in the booster vaccination group, it was unable to be matched by the reagent manufacturer and therefore not included in the Ct value analysis. There were no significant differences in minimum Ct values by age group and variant (Table 4).

**Table 4.** Minimum Ct values of Delta and Omicron variant infections stratified by age group and vaccination status.

		Delta variant		* *	* *	Omicron variant	* *	* *	* *
Age group (years)	Target	Primary vaccination ≥180 days median (IQR), N	Primary vaccination <180 days median (IQR), N	Booster dose median (IQR), N	*p* value	Primary vaccination ≥180 days median (IQR), N	Primary vaccination <180 days median (IQR), N	Booster dose median (IQR), N	*p* value
3–17	ORF1ab	-	24.0 [20.5;26.1]47	-	.	-	23.0 [22.0;25.0]33	-	.
	N	-	22.4 [18.5;25.0]47	-	.	-	23.0 [21.0;25.0]33	-	.
18–49	ORF1ab	22.0 [20.0;26.0]59	22.0 [19.8;25.0]52	24.0 [22.0;28.0]29	0.075	23.5 [22.2;27.0]6	23.0 [20.5;25.0]23	23.0 [23.0;23.0]1	0.690
	N	21.0 [18.0;25.0]59	21.0 [19.0;23.2]52	22.0 [20.0;26.0]29	0.298	24.0 [23.0;28.0]6	22.0 [20.0;25.0]23	24.0 [24.0;24.0]1	0.397
≥50	ORF1ab	21.0 [18.5;25.1]51	21.0 [18.0;23.9]95	21.8 [18.5;25.0]23	0.637	23.0 [22.0;25.0]5	26.5 [24.8;28.2]2	-	0.558
	N	20.0 [17.0;23.1]51	20.0 [17.0;22.1]95	22.0 [17.2;23.0]23	0.721	23.0 [22.0;24.0]5	26.0 [25.5;26.5]2	-	0.245
All	ORF1ab	21.7 [19.0;26.0]110	22.0 [19.0;24.1]194	23.7 [19.8;26.1]52	0.124	23.0 [22.0;26.5]11	23.0 [22.0;25.0]58	23.0 [23.0;23.0]1	0.873
	N	20.9 [18.0;24.3]110	21.0 [18.0;23.0]194	22.0 [18.0;24.2]52	0.452	23.0 [22.5;27.0]11	23.0 [21.0;25.0]58	24.0 [24.0;24.0]1	0.558

## Discussion

Our case-case study showed Omicron variant infection was 66% less likely to result in pneumonia than Delta variant infection. For Delta variant infections, compared with full primary vaccination at least six months before infection, full primary vaccination within 6 months of infection reduced the risk of getting pneumonia by 52%, and receipt of a booster dose reduced the risk of pneumonia by 82%. For Omicron infections, full primary vaccination within 6 months reduced the risk of getting pneumonia by 66%. There were too few Omicron cases in the booster group to calculate a valid odds ratio of pneumonia. We found no statistically significant association between viral load and vaccination status, although there was a trend toward lower viral loads with an increase in vaccination status.

There were no severe or critically-severe Omicron infections in this province-wide outbreak, and risk of pneumonia in all age groups and all vaccination statuses was lower among Omicron infections than Delta infections. These findings are consistent with reduced severity of Omicron cases in a study in a hospital in South Africa [12], in an England cohort study [13], and in a two-year observational study in southern Sweden [14].

The first six months following full primary series vaccination was associated with reduced risk of getting pneumonia and booster dose after six months of primary vaccination was associated with reduced risk of pneumonia in our study. This finding is consistent with an observational study of BNT162b2 mRNA COVID-19 vaccine [15] that showed that a third dose is effective in protecting individuals against severe COVID-19-related outcomes compared with receiving two doses at least 5 months before exposure to SARS-CoV-2.

That we found a non-statistically significant trend in the age-group analyses in minimum Ct value by vaccination status may reflect the small sample size in our study. The trend we found is consistent with a study showing that the decreased viral load after receipt of Pfizer’s mRNA vaccine began to diminish two months after primary series vaccination and did not persist after six months unless a booster dose was administered [18]. Overseas studies have shown that maximum viral loads in Omicron-infected patients are lower than in Delta-infected patients, but we did not find differences by variant. It is possible that natural infection after vaccination leads to higher neutralization antibody titres (hybrid immunity) than primary and booster immunity [19,20] and leads to viral load differences by variant. Since there were relatively few natural infections in China during or prior to our study, a lack of hybrid immunity could have led to the lack of difference in the maximum viral load between Omicron-infected patients and Delta-infected patients in our study.

In the epidemic spread in Henan, only 15% of the Delta-infected individuals were <18 years old. In contrast, 45% of the Omicron infected individuals were <18 years old. China vaccinated children <18 years old later than adults (starting mid-July 2021 versus December 2020), which may have confounded the relation between vaccination age and variant. Similarly, 16.1% of the Delta-infected individuals in our study received booster doses, while only 2.4% of the Omicron-infected people received booster doses. Since children were vaccinated later and during this study period were not recommended for booster doses, the difference is most likely related to timing of vaccination policy.

Our study has several strengths. The Delta and Omicron outbreaks were simultaneous and in a single province of China, allowing differences in Delta and Omicron infections to be observed in similarly-vaccinated populations. The nearly-complete lack of previous local transmission in Henan means that immunity in our study was entirely vaccine-induced and not hybrid immunity. This allowed comparison of vaccine effectiveness against pneumonia and viral load by the two variants in a level playing field of pure vaccine-induced immunity.

Our study has limitations. First, our study was based on cases only (a case-case study), and so the results are not vaccine effectiveness against pneumonia, but an estimation of the association between vaccination status and pneumonia. The association is a measure of impact on top of an unmeasured baseline vaccine effectiveness. The design we used has been used in vaccine effect evaluations for influenza vaccines [10, 16] and COVID-19 vaccines [17]. Second, because the numbers of people infected with Delta and Omicron strains were small, the 95% confidence intervals were wide and we were unable to conduct desirable subgroup analyses such as relative VE by specific comorbidity. The comorbidities in our subjects were high blood pressure, diabetes, cerebral vascular disease, coronary heart disease, asthma, emphysema, chronic bronchitis, lung cancer, chronic liver disease, liver cancer, chronic kidney disease, immunodeficiency, AIDS, tuberculosis, and pregnancy. Third, there were only 10 Omicron cases that received a booster dose. Therefore, we could not evaluate ORs of booster dose associated with pneumonia among Omicron infections. Fourth, collection of Ct values was not part of a predetermined study design, and so the number of nucleic acid tests, reagent manufacturers, and testers varied. Although our study used one of the most commonly used reagents, Ct values can also be affected by the extraction technology and the test personnel [21]. The number of samples included in Omicron analysis was small (70 in total), thus affecting the stability of the minimum Ct value by vaccination status in Omicron patients.

In conclusion, our analysis of the association between vaccination status and pneumonia among Delta and Omicron infections in a Henan province outbreak found that Omicron infection is less likely to lead to pneumonia than Delta infection. The first six months after primary vaccination with China-produced vaccines and administration of booster doses after six months were associated with much lower risk of pneumonia. We found no difference in Ct values by vaccination status or variant. We recommend continuing to increase primary series coverage and accelerating use of timely booster doses, six months after primary vaccination. Monitoring vaccine use, safety, and effectiveness by vaccine type and schedule is essential for keeping COVID-19 vaccination policy up-to-date and as effective as possible.
